# COVID-19 and Liver Surgery: How the Pandemic Affected an Italian Medium-Volume HBP Center

**DOI:** 10.3389/fsurg.2022.918348

**Published:** 2022-06-28

**Authors:** Francesca Carissimi, Mauro Alessandro Scotti, Cristina Ciulli, Alessandro Fogliati, Fabio Uggeri, Marco Chiarelli, Marco Braga, Fabrizio Romano, Mattia Garancini

**Affiliations:** ^1^ Department of Surgery, HPB and Gastroenterological Surgery Unit, San Gerardo Hospital, Monza, Italy; ^2^ Department of Surgery, School of Medicine, University of Milano-Bicocca, Milan, Italy; ^3^ Department of Surgery, Manzoni Hospital, Lecco, Italy

**Keywords:** COVID-19, liver surgery, liver disease, pandemic, laparoscopy

## Abstract

**Introduction:**

While the COVID-19 pandemic is still ongoing, it is even more evident that victims of the pandemic are not only those who contract the virus, but also the countless patients suffering from other serious diseases (i.e., tumor) who have undergone delayed potentially life-saving surgery due to a lack of beds. Like many hospitals, ours also initially blocked all elective oncologic surgery, but these operations were “recovered” and reintegrated in a relatively short time, thanks to the establishment of COVID-free wards and operating rooms with staff dedicated to oncological surgery. In tis context, our aim is to assess whether and how the severe acute respiratory syndrome Coronavirus-2 (SARS-CoV-2) pandemic has impacted our hepatobiliary surgery unit.

**Methods:**

From our prospective database, we retrospectively took data from patients undergoing liver surgery in 2018–2019 (pre-COVID) and 2020–2021 (COVID period). Patients admitted to COVID-free wards must necessarily have a negative nasal swab from the previous 24 h.

**Results:**

Between January 1, 2018, and December 31, 2019 (Group 1), 101 patients were treated; during the pandemic [January 1, 2020, and December 31, 2021 (Group 2)], 126 patients were treated. There was no statistical difference between the groups. The median postoperative hospital stay was 7 days for both groups; 7 patients had major complications (Clavien-Dindo > 3) in Group 1 and 11 in Group 2 (*p* = 0.795). A total of 4 patients died in Group 1 and 6 during the pandemic (*p* = 0.754). Tumor burden was significantly greater in Group 2 where nodule size, lymphadenectomy, and extrahepatic disease were significantly greater (*p* = 0.011, *p* = 0.004, and *p* = 0.026, respectively).

**Conclusion:**

During the COVID pandemic, our HPB unit managed to offer a volume of tertiary-center hepatobiliary surgery without a significant impact in terms of length of stay, morbidity, or mortality despite the increase in tumor burden during the pandemic years.

## Introduction

The severe acute respiratory syndrome Coronavirus-2 (SARS-CoV-2) has spread globally since the end of December 2019, exponentially increasing the need for intensive care of the affected population; this growth has focused health forces on new interstitial pneumonia (1, 2). Italy, in particular the Northern region, was the European nation to suffer the most from the devastating effects of COVID-19, with mortality peaks much higher than the European median (over 15 daily deaths per million inhabitants) (3, 4). To ensure access to care for all patients with SARS-CoV-2 pneumonia, profound changes were made in hospital activities to redistribute resources with a consequent radical decrease in outpatient visits and surgical interventions (5). Follow-up and non-urgent visits were suspended, as were all routine diagnostic tests. Surgery was reorganized guaranteeing only elective cancer surgery and emergency surgery (6–8). Neoplastic liver diseases were also affected by the pandemic; in fact, outpatient visits were drastically reduced due to the lack of human resources and the reluctance and fear of patients to go to hospital, causing a delay in the diagnosis of primary cancers or relapses.

Cancer patients have, therefore, become a category affected by COVID-19 without being infected due to the delay in diagnosis and the consequent progression of the disease, worsening the long-term outcome of these patients. Furthermore, when the operating rooms were reactivated for cancer patients, the infection (even asymptomatic or paucisymptomatic) represented a contraindication to the planned intervention due to the possible liver damage related to the COVID infection (9) which would complicate the already delicate post-operative course. COVID-19 infection could be responsible for the development of post-hepatectomy liver failure (PHLF), and SARS-CoV-2-related pneumonia after liver surgery could be an independent risk factor for morbidity and mortality (10). PHLF is the most life-threatening complication after liver surgery (11, 12). Only a very few papers have focused on the effect of COVID-19 on liver surgery practice. We report here the experience of our medium-volume Liver Surgery Center (13). The aim of the study is to evaluate the effects of the COVID-19 pandemic on liver surgery in a middle-volume Italian Center.

## Methods

We retrospectively analyzed the data collected from a prospectively maintained database of all patients undergoing liver surgery at the HPB Unit of the San Gerardo Hospital, Monza, University of Milano Bicocca from January 1, 2018, to December 31, 2021.

According to Torzilli et al. (13), we are considered a middle-volume center for liver surgery. Patients were divided into two groups: Group 1 (January 1, 2018, to December 31, 2019) before the pandemic and Group 2 (January 1, 2020, to December 31, 2021) during the pandemic.

Every patient who underwent elective surgery in 2020–2021 was screened for COVID-19; if any patient was affected by SARS-CoV-2, surgery was always delayed until the resolution of COVID-19. During hospitalization, patients underwent control nasal swabs on days 3–5–10 and every week until discharge. If a patient became positive at control swabs, he was immediately transferred to the COVID ward and his roommates placed in isolation.

A comparative analysis of the two periods was conducted to evaluate the effects of the COVID-19 pandemic on liver surgery; preoperative data and surgical and postoperative outcomes were analyzed and compared between study periods.

Clavien–Dindo classification (14) and the Comprehensive Complication Index (CCI) (15) were used to assess the complication rate in our population. Statistical significance was accepted at *p* < 0.05. The study was conducted in accordance with the regulations for retrospective studies of our hospital.

Data analysis was performed using IBM SPSS Statistics. Statistical analysis was obtained for the main descriptive indexes. Quantitative data were expressed as mean ± standard deviation (SD) or median and interquartile range (IQR) depending on the statistical appropriateness. The qualitative data were elaborated as absolute frequencies, relative frequencies, cumulative frequencies, and percentages.

## Results

From January 2018 to December 2019 (Group 1), a total of 101 patients were treated in our HPB unit, and from January 2020 to December 2021 (Group 2), the total number was 126; Table 1 shows that there was no alteration in the patient baseline, decision making, and quality of care as there was no statistical difference in patient demographics, indications for surgery, and complexity of procedures.

**Table 1:** Baseline of Group 1 and Group 2.

	Group 1 (*n* = 101)	Group 2 (*n* = 126)	*p*
Age (years)	68 ± 10	67 ± 11	0.659
Sex (M)	69 (68.3)	71 (56.3)	0.065
Charlson comorbidity Index	8 ± 2	7 ± 2	0.378
Diabetes	25 (24.7)	18 (14.2)	0.160
Renal disease	9 (8.9)	6 (4.7)	0.423
Cardiovascular disease	14 (13.8)	10 (7.9)	0.310
Respiratory disease	6 (5.9)	6 (4.8)	0.929
*Liver disease*			*0* *.* *076*
None (healthy liver)	41 (40.7)	61 (48.4)	
Child-Pugh A5	33 (32.8)	35 (27.7)	
Child-Pugh A6	17 (16.8)	21 (16.7)	
Child-Pugh B7	7 (6.9)	5 (3.9)	
Child-Pugh B8	2 (1.9)	3 (2.5)	
Child-Pugh B9	1 (0.9)	1 (0.8)	
MELD-Na score	7 ± 3	8 ± 2	0.107

*Baseline of Group 1 and Group 2, Child-Pugh Score, and MELD-Na Score were calculated for patients with underlying liver disease.*

Mainly liver resection in the two groups was done for HCC and liver metastases; Table 2 shows in detail the pathologies treated. Most of the interventions were segmentectomies and atypical liver resections with similar rates in the two groups; in Group 1, 4% of patients underwent major resection [3 or more contiguous liver segments (16)], whereas in Group 2, this percentage rose to 7% without reaching statistical significance with a *p* of 0.476 (Table 3).

**Table 2:** Liver pathologies treated pre-COVID (Group 1) and during pandemic (Group 2).

	Group 1	Group 2	*p*
Hepatocellular Carcinoma	51 (50.5)	65 (51.7)	0.213
Metastasis.	34 (33.7)	37 (29.4)	
CRLM	25 (73,5)	29 (78.4)	
Other	9 (26.4)	8 (21.6)	
Cholangiocarcinoma	7 (6.9)	15 (11.8)	
Benign	9 (8.9)	9 (7.1)	
Total	101 (100.0)	126 (100.0)	

*CRLM: Colorectal liver metastasis; Other metastasis included: Gastric Cancer, Ovarian Cancer, Renal cancer, NET (Neuroendocrine Tumor), Breast Cancer, and Melanoma. The percentages are referred to the total number of metastases.*

**Table 3:** Type of surgery and extension of liver resection.

	Group 1 (*n* = 101)	Group 2 (*n* = 126)	*p*
ALPPS	1 (1.0)	2 (1.7)	0.790
Bisegmentectomy	10 (9.7)	15 (11.9)	
Right hepatectomy	1 (1.0)	4 (3.2)	
Left hepatectomy	2 (1.9)	3 (2.4)	
Parenchymal sparing Resection	61 (60.8)	65 (51.7)	
Segmentectomy	14 (13.9)	16 (12.6)	
Subsegmentectomy	3 (2.9)	8 (6.3)	
Cyst fenestration	7 (6.9)	5 (3.9)	
Termoablation only	2 (1.9)	8 (6.3)	
Major	4 (3.9)	9 (7.2)	0.476
Minor	94 (93.1)	115 (91.2)	
Not-therapeutic Laparotomy	3 (3.0)	2 (1.6)	

*Major liver resections include 3 or more contiguous liver segments according to Brisbane.*

*Classification (* *16* *). ALLPS: Associating Liver Partition and Portal vein ligation for Staged hepatectomy.*

The tumor burden in the group of patients treated before the pandemic was 2 ± 2 nodules, but during the pandemic, the mean increased to 2 ± 2 nodules. The size of the major nodule increased from a median of 21 (IQR 15–30) in Group 1 to 25 (IQR 11–28) in Group 2 with a statistically significant difference (*p* = 0.011). We also noticed in patients affected by cholangiocarcinoma and metastases an increase in lymphadenopathy and extrahepatic disease from 6 to 16 and from 9 to 21 in the two groups, respectively, with a *p* of 0.004 and 0.026, respectively. During cholangiocarcinoma surgery, we performed lymphadenectomy, and the number of lymph nodes collected in Group 1 was 3 (IQR 0–5), and in Group 2, it was 4 (IQR 2–6) with *p* = 0.361; of these, the number of metastatic lymph nodes increased from 1 (0–1) to 3 (1–5) between two study periods (*p* = 0.015). The length of surgery was 277 ± 129 min in Group 1 and 360 ± 162 min in Group 2 with no difference; during the pandemic, the number of intraoperative RF treatments increased from 2 (Group 1) to 14 (Group 2) with a *p* of 0.008. The laparoscopic approach was employed in 44.6% of patients in Group 1 and in 59.5% in Group 2, with a significant increase (*p* = 0.049); the conversion rate was 31.1% and 22.6%, respectively, in Group 1 and Group 2 (*p* = 0.127). The laparotomy approach did not change significantly (*p* = 0.09) in the two groups: 55.4% in Group 1 and 40.5% in Group 2 (Table 4).

**Table 4:** Comparison of tumor burden between Group 1 and Group 2.

	Group 1 (*n* = 101)	Group 2 (*n* = 126)	*p*
N° nodules	2 ± 2	2 ± 2	0.247
Size of major nodule (mm)	21 (15–30)	25 (11–28)	**0.011**
Macrovascular Invasion	2 (1.9)	4 (3.2)	0.440
Lymphadenopathy	6 (5.9)	16 (12.7)	**0.004**
N° of retrieved lymphnodes	3 (0–5)	4 (2–6)	0.361
N° of metastatic lymphnodes	1 (0–1)	3 (1–5)	**0.015**
Extrahepatic disease	9 (8.9)	21 (16.7)	**0.026**
Intraoperative RF	2 (1.9)	14 (11.1)	**0.008**
Operative time (min)	277 ± 129	360 ± 162	0.280
Laparoscopic approach	45 (44.6)	75 (59.5)	**0.049**
Conversion rate	14 (31.1)	17 (22.6)	0.127
Laparotomic approach	56 (55.4)	51 (40.5)	0.09

*Lymph nodes were retrieved for patients with cholangiocarcinoma.*

*The bold valueas are the significant ones (p < 0.05).*

The postoperative hospital length of stay was 7 (IQR 5–11) days before the pandemic and 7 (IQR 5–17) during the pandemic (*p* = 0.183). The complication rate was comparable in the two groups, as shown in Table 5; the major complications (CD >3) were 7.9% and 8.7% in Groups 1 and 2, respectively (*p* = 0.795), four patients died during the prepandemic period and six died during the pandemic (*p* = 0.754). The CCI demonstrated no significant differences between the two groups, 0.0 (IQR 0–20.9) in Group 1 vs. 8.7 (IQR 0.0–24.2) in Group 2 (*p* = 0.559). No patient developed COVID-related pneumonia or became positive when the control nasal swab was applied during hospitalization.

**Table 5:** Post-operative course and complication.

	Group 1 (*n* = 101)	Group 2 (*n* = 126)	*p*
Post-operative LOS [day]	7 (5–11)	7 (5–12)	0.183
Complications No	51 (50.5)	54 (42.9)	0.795
Minor (CD 1–2)	29 (28.7)	36 (28.6)	0.795
Major (CD 3a–4b)	8 (7.9)	11 (8.7)	0.795
Death	4 (3.9)	6 (4.7)	0.754
Comprehensive Complication Index	0.0 (0–20.9)	8.7 (0.0–24.2)	0.559

*LOS, length of stay, CD, Clavien–Dindo Classification for surgical complications* (*14*)*.*

## Discussion

The COVID pandemic put a strain on the Italian health system, which suddenly found itself facing several critically ill patients for whom there were insufficient resources (17). A shortage of healthcare professionals and limited bed capacity were major hurdles in the care of patients with oncological disease during the pandemic. To tackle this problem, HUB hospitals were identified to refer patients affected by COVID-19, including our center. A large part of medical staff was recruited into COVID departments due to this reorganization; this choice caused problems in the management of all other wards and activities. The unusual, altered allocation of healthcare resources might be responsible for collateral damage to oncological patients: screening interruption, treatments deleted or downgraded, and follow-up delay (18). Moreover, cancer patients are at major risk to contract COVID-19 infection both from nosocomial exposure and from their immunocompromised states (19). In our Liver Surgery Unit, all cases are discussed in multidisciplinary meetings and are managed following European Association for the Study of the Liver (EASL) guidelines and the Barcelona Clinic Liver Cancer (BCLC) staging system (20, 21). From our experience, we noted a reduction in hepatic procedures during the first and second pandemic wave (Group 1), with an increasing rebound in Group 2, during which hospital resources were organized to deal with COVID but to maintain activity for oncologic patients. The multidisciplinary group, during the first period of the pandemic, had suspended the meetings to avoid contagion of the involved specialists; moreover, the possibility of treating the patients already discussed was almost impossible due to the suspension of the elective activity—albeit short—of the first wave. Furthermore, the absence of outpatient clinics and follow-ups did not allow the enrollment of new patients. Obviously, specialists have always had the opportunity to communicate with one another telematically if necessary, and as soon as it was possible, multidisciplinary discussion was reintroduced. Overlooking our data, we could see how the number of patients treated during the COVID pandemic (Group 2) did not decrease, but rather increased even if in a not statistically significant way (*p* 0.213); this demonstrated how the impressive rearrangement of the health system permitted to maintain a high level of assistance for patients undergoing liver surgery (4).

With the gradual resumption of normal activities since January 2020, we have had a rebound in the number of patients and outpatient visits due to the waiting lists caused by the blockade during the pandemic.

The resumption of visits led to the diagnosis of new tumors in patients who were not followed up. Our data, in agreement with those of the existing literature (22, 23), did not show statistically significant differences in the number and type of liver disease treated (*p* = 0.213).

However, upon analyzing our data, what emerged was an increase in tumor burden, with an increase in the size of nodules (*p* = 0.011), lymphadenopathy (*p* = 0.004), and extrahepatic disease (*p* = 0.026) in patients treated after the pandemic (Group 2); in a recent study from six academic referral centers in France, the authors reported a delay longer than one month in the diagnosis and treatment of HCC in the pre-COVID era, and the COVID period was found to be a strong independent predictor of treatment delay or cancellation (24). This could justify the presence of an increased tumor burden at diagnosis compared with the pre-COVID era (Group 1). As demonstrated by our experience, tumor progression occurred in patients with more aggressive tumor biology during the pandemic due to delayed or discontinued follow-up and treatment, and the consequence was a shift toward a higher tumor burden at diagnosis (25). Furthermore, cirrhotic patients may be more prone to hepatic decompensation during the period of social lockdown due to the lack of controls, and the worsening of liver function in these cases has made the HCC management plan more complex in case of cirrhosis (26).

Tellez et al. (27) show how the COVID-19 pandemic had a huge impact on the routine care of liver cancer patients; what can be seen from our data is the increase in patients who underwent intraoperative radiofrequency (as a single treatment strategy or associated with liver resections) during the pandemic (*p* = 0.008) due to a greater tumor burden, which made the technically more complex or impossible surgical resection possible (i.e. vascular invasion at the IOUS). Balakrishnan et al. (28), in their survey, show how this change in therapeutic strategy has occurred worldwide. Internationally, HPB centers have reported a reduction in the number of surgical resections and the adoption of a non-surgical management of pathologies that were traditionally treated surgically, although the Society of Surgical Oncology (29) asserts that the surgical approach is recommended for aggressive hepatobiliary malignancies, also stating that neoadjuvant chemotherapy, ablation therapies, or stereotactic radiotherapy could be considered for resectable liver metastases or HCC. The Italian Association for the Study of the Liver conducted a survey to evaluate the impact of COVID-19 on the activity of some hepatology units, reporting that the surgical treatment of HCC had been significantly reduced or even stopped (30, 31).

The shift of liver cancer therapy toward nonsurgical treatment is justified by a lower complication rate associated with a good outcome in oncological terms for the patient (32). The use of intraoperative radiofrequency impacts less on the post-operative course by requiring a stay in intensive care in a very limited number of cases. This is a key issue in the COVID era due to the limited number of beds in ICU; and radiofrequency also reduces the post-operative hospital stay by reducing the patient’s risk of contracting the virus (33).

Liver resection during a pandemic should be limited to: (i) patients with a low risk of liver decompensation; and (ii) those without comorbidities that increase the risk of severe COVID-19 (34). Since January 2020, the COVID-19 pandemic has led to several debates regarding different aspects of the management of the surgical patient, especially related to the possible risk for healthcare professionals during surgery (35). The use of minimally invasive surgery when an indication for hepatic resection has been established is to be preferred due to its advantages such as a decreased length of stay, reduced postoperative complications, and, in general, less need for treatment (36).

The possible aerosolization of viral pathogens during laparoscopy is still controversial (37, 38). It is essential that all staff in the operating room wear personal protective equipment and use the AIRSEAL® system as suggested by the SAGES-EAES Guidelines (39) for gas recirculation.

Following these pieces of advice, we do not reduce laparoscopic approach to liver surgery in the pandemic period; instead, we increase significantly (*p* = 0.049) the number of laparoscopic procedures to be performed.

Postoperative complications did not show differences between our two groups in terms of LOS, major complications, and death (*p* > 0.05). Our results differ from the survey, and based on the IHPBA-COVID Registry, reported that COVID-19 was associated with a high mortality rate after hepato-pancreato-biliary (HPB) surgery (29% for pancreaticoduodenectomy, 15% major hepatectomy, and 3% cholecystectomy); moreover, cancer patients are at major risk to contract COVID-19 infection both from nosocomial exposure and from their immunocompromised states (19). Fortunately, none of our patients developed SARS-CoV-2 infection, thanks to our prevention measures aforementioned: dedicated COVID-19-free areas of the hospital (ward and operating room); (ii) healthcare personnel dedicated only to COVID-19-free areas, using protective equipment; and (iii) periodic nasal swab to all patients to confirm negativity or identify early positivity in order to protect frail patients.

## Conclusion

COVID has had a great impact on patient care activities due to the profound readjustment of the health system to address the new needs dictated by the pandemic. Liver cancer patients have seen their follow-up postponed or even deleted with later diagnosis, which has led to a greater tumor burden, with a consequently different curative approach to the pathology (an increase in thermoablative treatments). Besides, what we have learned from the pandemic is that a rapid and prompt reorganization of activities, focused on cancer patients, does not affect the type of surgical approach or the patient’s outcome; in fact, despite all changes, the complication rate remains stable in our HBP unit.

## Data Availability

The raw data supporting the conclusions of this article will be made available by the authors, without undue reservation.
